# Effects of fatty acids and cholesterol on functions and behavior of bone marrow mesenchymal stem cells

**DOI:** 10.1016/j.isci.2026.114813

**Published:** 2026-02-10

**Authors:** Blanca Gonzalez-Garcia, Miriam Plaza, Maria Fresco, Cristina Aparicio, Rocio Abia, Francisco J.G. Muriana, Sara M. Jaramillo-Carmona

**Affiliations:** 1 Instituto de la Grasa, The Spanish National Research Council (CSIC), Laboratory of Cellular and Molecular Nutrition, Seville, Spain

**Keywords:** Stem cells research

## Abstract

Bone marrow (BM) mesenchymal stem cells (BM-MSCs) have a crucial role in BM homeostasis and regenerative therapy. While lipids are known to be fundamental components of metabolism, their precise role in modulating the function and behavior of BM-MSCs remains unclear. In this review, we present a comprehensive update on the biology of BM-MSCs in response to fatty acids and cholesterol, which are the major health-related lipids. We discuss the importance of BM as a receptor and producer of lipids and summarize the mechanistic insights by which cholesterol and fatty acids, including endocannabinoids and short-chain fatty acids, regulate BM-MSC stemness, rejuvenation, and commitment. Understanding the complex and interdependent role of lipids on BM-MSCs is critical for proper BM and bone homeostasis. Future research should focus on the development of metabolic strategies to optimize health of the BM and its cells and to refine treatments for BM-related diseases or tissue regeneration.

## Graphical abstract

**Figure:**
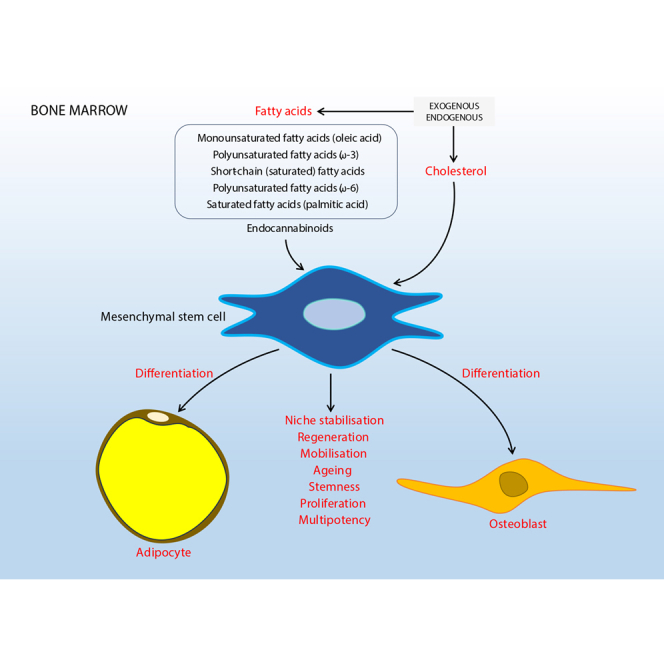

## Introduction

Mesenchymal stem cells (MSCs) are a population of adult multipotent progenitor cells with the ability of self-renewal and multiple lineage differentiation.^1^ These cells were first isolated from bone marrow (BM) aspirates in 1966.^2^ In the last few decades, MSCs have come to the forefront of regenerative medicine field due to their anti-inflammatory and immunomodulatory properties, as well as their regenerative potential to replace a wide variety of terminal cells in damaged tissues.^3^ In fact, a PubMed search for “mesenchymal stem cell therapy” returned 51,531 results (May 2025), with an exponential increase since 2008. This number represents approximately half of research that has been carried out on the topic “mesenchymal stem cells.” In addition, the same search on clinicaltrial.gov returned 712 clinical trials, either complete or incomplete. According to the International Society for Cellular and Gene Therapy (ISCT), MSCs must meet three basic criteria before they can be used.^4^ Firstly, they must be plastic-adherent in standard culture. Secondly, they must express cluster of differentiation 73 (CD73), CD90, and CD105, while being devoid of CD45, CD34, CD14, CD19, and human leukocyte antigen DR (HLA-DR) surface molecules. Thirdly, they must be able to differentiate into osteoblasts, adipocytes, and chondroblasts under appropriate culture conditions. Despite this, an overwhelming amount of research on MSCs has been published recently, highlighting the lack of complete consensus on the criteria for their identification and successful application. In turn, not only have other groups proposed additional markers or functional characteristics for MSC identification, but the ISCT has also reiterated the need to expand its defining criteria to include the tissue of origin, functional definitions, and a matrix of functional assays when talking about MSCs for safe and appropriate therapeutic use.^5^^,^^6^^,^^7^

BM is one of the most widely used sources of MSCs (BM-MSCs), and others include white adipose tissue, umbilical cord, placenta, endometrium, skin, dental pulp, and connective tissue, although several studies suggest that MSCs can be found in virtually all tissues as they can invade and spread within blood vessels.^8^^,^^9^^,^^10^^,^^11^ There may be small but significant differences between MSCs in terms of their active role in supporting tissue homeostasis, despite this wide range of anatomical locations.^12^ Due to their environment within the bone, BM-MSCs play a key role in regulating hematopoiesis, a process by which hematopoietic stem cells (HSCs) continuously differentiate to replenish all blood cell types.^13^ Hematopoiesis is a vital process that maintains homeostasis during the development of many physiological functions, such as the immune system, coagulation, and oxygen transport. Therefore, a tight regulation of BM-MSCs is the key to maintaining a healthy BM and overall health.^14^

Beyond their direct interactions with and inherent multipotency close to that of HSCs, BM-MSCs also contribute to the formation of other relevant components of the BM microenvironment. A particularly interesting aspect of this process is the formation of the BM adipose tissue (BMAT), which consists of a dispersed configuration of single cell adipocytes without a collagen support system—a key feature that arises after *in situ* differentiation of BM-MSCs.^15^ BMAT is a specialized type of adipose tissue whose functional properties are not yet fully understood and that differs significantly from the adipose tissue found in other areas of the body. Recent research suggests that BMAT may have a dual role—energy storage in the form of triglyceride-rich BM adipocytes and the active regulation of hematopoiesis and bone remodeling as a result of osteoblastic differentiation of BM-MSCs—processes in which BM adipocyte lipids may be important players.^16^^,^^17^^,^^18^^,^^19^ However, these complex and interdependent relationships are still poorly understood, and there are still major gaps in the biology of BM-MSCs, particularly those residing in the bones where they coexist with their differentiated progeny. These include how their metabolic activity is influenced by lipids, either dietary or endogenous, such as fatty acids and cholesterol,^20^^,^^21^^,^^22^ and then, or in the meantime, the cellular and molecular mechanisms that regulate their intramedullary proliferation and fate, and perhaps their therapeutic utility. Thus, the aim of this review was to update the current state of knowledge and briefly present new perspectives on the function and behavior of MSCs, mainly in the BM compartment, in response to exogenous and endogenous lipids. The focus was on cholesterol and fatty acids as the most prominent lipids involved in health. We discuss the possible underlying mechanisms and biological pathways, and we also attempt to reflect on some of the calls for future investigation.

## BM receives and produces lipids

The BM is enclosed in the inner part of long bones (humerus, femur, and tibia), flat bones (sternum and ribs) and short bones (bones of the skull).^23^ This tissue unit is composed of a complex tapestry of HSCs (hematopoietic progenitors and mature cells of myeloid and lymphoid lineages) and non-hematopoietic cells (MSCs, non-hematopoietic progenitors, adipocytes, osteoblasts, neurons, vascular cells, among others).^24^ These components are not evenly distributed within the BM but are heterogeneously distributed as they are organized into specialized microenvironmental niches.^25^ At least two major anatomical niches can be found in the BM, depending on the distance from the bone tissue: the endosteal niche, close to the bone surface, and the central niche, in the inner BM. In addition, like the rest of biological tissues, the BM contains a multitude of blood and lymphatic vessels responsible for supplying it with oxygen and nutrients, as well as a pleyade of chemical signals in the form of hormones, metabolites, and exosomes, to name but a few.^26^^,^^27^ Arterioles branch into capillaries in the metaphysis or into sinusoids in the diaphysis of the bone (also known as type H and type L vessels).^28^^,^^29^^,^^30^ While the former are characterized by a continuous wall, the latter are fenestrated vessels that provide high permeability to the central niche. The interconnected capillaries and sinusoids then converge on the central vein.^31^ This extensive network of blood vessels makes the BM a major recipient of blood components, such as circulating lipids, as well as a sender of soluble components in the BM milieu to the blood. These vessels also act as a vascular highway, enabling blood cells and BM-MSCs to move back and forth between the BM and the blood.

As far as exogenous lipids as nutrients are concerned, triglycerides are the dominant lipids in the diet, accounting for 90%–95% of the total energy derived from dietary fat. Cholesterol, phospholipids, and many other lipid molecules, such as fat-soluble vitamins, are also part of dietary fats. After triglyceride digestion, once fatty acids and 2-monoacylglycerols are absorbed in the small intestine by protein-independent diffusion models and protein-dependent mechanisms, and cholesterol is absorbed by Niemann-Pick-type C1-like 1 protein, most of dietary fatty acids (mainly in the form of triglycerides after resynthesis using 2-monoacylglycerols as the starting hydrocarbon backbone) and, to a lesser extent, cholesterol are assembled in the enterocytes and secreted into the systemic circulation as the lipoprotein form of chylomicrons.^32^ Dietary fatty acids and cholesterol can then reach every corner of the BM on a daily basis, where BM-MSCs are enriched in hypervascularized areas,^33^ receiving up to 10% of total cardiac output and providing a conduit for the enormous energy demands of the human skeleton. However, there may be different contributions to BM cell maintenance and potency of stem cells, including BM-MSCs, depending on the lipid nutrient (and most likely its amount).

**Figure 1:**
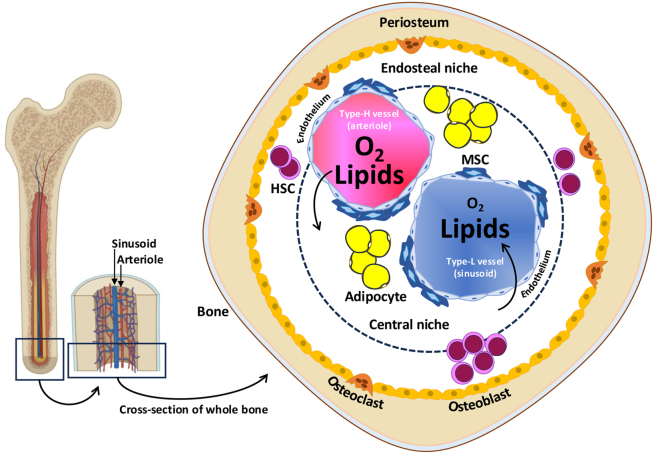
Simplified overview of the bone marrow (BM), focusing on the major blood vessels that supply oxygen (in erythrocytes) and lipids (in lipoproteins) from the bloodstream, and on the expected localities of the stem cells of hematopoietic and mesenchymal origins (HSCs and MSCs, respectively), including the progeny (osteoblasts and adipocytes) of MSCs The partial pressure of oxygen is highest at the entry of the arteriole into the BM and decreases with its use in the respiratory system of the cells, while lipids are expected to be similar in amount at the exit of the sinusoid by a potential balance of inputs and outputs under normal conditions. The figure was partially realized with www.biorender.com.

Another exciting piece of the puzzle is endogenous fatty acids and cholesterol, which result from lipid metabolism in the BM during starvation. BMAT is the primary neutral lipid reservoir in the BM. However, other BM cells, such as BM-MSCs, osteoblasts, and osteocytes, are able to accumulate lipid droplets, mostly in association with disease or metabolic disorders.^33^ It is well known that BMAT increases during starvation, while extramedullary adipose tissue, especially the white adipose tissue, decreases. This supports the idea that BMAT has an evolutionary role of being the final reserve of energy storage, confined to the BM. When catabolism is activated in a catecholamine-dependent or -independent manner, the main mechanism for the release of fatty acids into the BM environment is the lipolysis of triglycerides by canonical and alternative non-canonical pathways in the BM adipocytes, in which adipose tissue triglyceride lipase plays a central role as the rate-limiting step.^34^^,^^35^ In addition, cholesterol can be found in the BM mainly through the presence of cholesterol-rich lipoproteins (low-density lipoproteins) after processing of very-low-density lipoproteins in the bloodstream and from endogenous metabolism of BM-resident cells. Cholesterol can also be detected in the BM as a pro-regenerative metabolic signal carried in very-low-density lipoproteins from the liver,^36^ which is the tissue for synthesizing the majority of cholesterol in the body (producing three times more than is present in a healthy diet) and maintaining cholesterol homeostasis.^37^ It is interesting to note that if BMAT does not have the ability to break down triglycerides, then it can acquire a metabolism that is orientated toward cholesterol.^38^ In this case, BMAT would also be expected to become a site of cholesterol storage and thus a potential transient local source of cholesterol in the BM. These lines of evidence highlight the close relationship between cholesterol and fatty acids within the BM and the potential importance and regulatory role that this combinatorial entity may play in any BM niche (Figure 1).

## How cholesterol at the interphase of fatty acids can affect MSCs in the BM

BM-MSCs are clustered in the central niche, which represents around 90% of the total BM area. They have a supportive, oxygenated environment, and their bioenergetics depend on the tricarboxylic acid (TCA) cycle, which is coupled to the electron transport chain (ETC)-oxidative phosphorylation (OXPHOS) system. This is in contrast to the endosteal niche, where hypoxic conditions favor the maintenance of BM-HSCs via glycolytic pathways.^39^ Detailed imaging studies have shown that BM-MSCs preferentially remain in the central niche as a subset of resident cells called Nestin-GFP^lo^ LEPR^+^ CXCL12-abundant reticular BM-MSCs, which adhere to localized areas close to the sinusoidal endothelium, or Nestin-GFP^hi^ NG2^+^ BM-MSCs, which adhere to vessels and arterioles at the endosteal transition zone.^13^^,^^40^ It is this proximity to the vasculature that allows BM-MSCs to access and respond to all blood components.

For example, the osteoblastic differentiation capacity of BM-MSCs has been shown to be imbalanced in favor of adipocyte production in the BM when excessive amounts of cholesterol are present in the diet of experimental animals,^33^^,^^41^ probably through mechanisms involving activation of the peroxisome proliferator-activated receptor γ (PPARγ) and inhibition of the Wingless-related integration site signaling pathway.^42^ This may explain why therapeutic doses of cholesterol-lowering drugs promote osteogenic differentiation while inhibiting the adipogenic differentiation of murine and human BM-MSCs.^43^^,^^44^^,^^45^ Similarly, abnormal self-renewal and differentiation of BM-MSCs resulting in osteoblast depletion were recently shown to be beneficially modulated by atorvastatin treatment in a rodent model of steroid-induced osteonecrosis of the femoral head,^46^ also establishing a potentially relevant role for cholesterol homeostasis in the signature of BM-MSCs within the BM.

An intriguing question is whether the dietary cholesterol-induced increase in BMAT is in itself harmful to the bones and BM, or whether it is a yet-to-be understood consequence, as a compensatory mechanism, limited to the cholesterol-induced behavior of BM-MSCs or to the BM-MSC-mediated accommodation of cholesterol in the BM microenvironment. In short, the BM adipocyte is commonly thought of as either a mere endocrine mediator between BM cells, such as hematopoietic and bone remodeling cells,^47^ or a troublesome occupant of space required by hematopoietic lineage cells.^13^ We argue that the concept of adversity in relation to BMAT enlargement is an interpretive paradigm. In order to better understand the role of BM adipocytes in the interrelationships of adult BM cell populations, the fatty acid composition of triglycerides in their adipocytes, including cholesterol, should be considered a significant factor.^48^^,^^49^^,^^50^ BM-MSCs, which are presumably in a naive and adherent state in the BM,^51^ can exert a pronounced long-term paracrine function. This is achieved by the secretion of soluble factors and the release of extracellular vesicles such as exosomes and microvesicles,^52^ whose cargo has valuable therapeutic potential for many diseases, including neurological disorders.^53^ This cargo is encapsulated in endosomal bilayer membranes that require large amounts of cholesterol. Caveolae, membrane invaginations in lipid rafts of BM-MSCs, also contain significant amounts of cholesterol,^54^ the levels of which are regulated by caveolin-1 (the major protein component of caveolae) to promote homeostasis and adhesion in the cytoplasmic membrane of human BM-MSCs.^55^ Age-related changes associated with the reactive oxygen species (ROS)/p53/p21^Cip1/Waf1^ signaling pathway and autophagy processes have been documented to be delayed by cholesterol in murine BM-MSCs.^56^ The dependence of BM-MSCs on cholesterol requires further research attention. This led us to hypothesize that the BM, as a microenvironmental cholesterol site, and BMAT, as an additional cholesterol reservoir, act as local sources to maintain the intense autocrine and paracrine activity and rejuvenation of BM-MSCs, even in elderly individuals.

**Figure 2:**
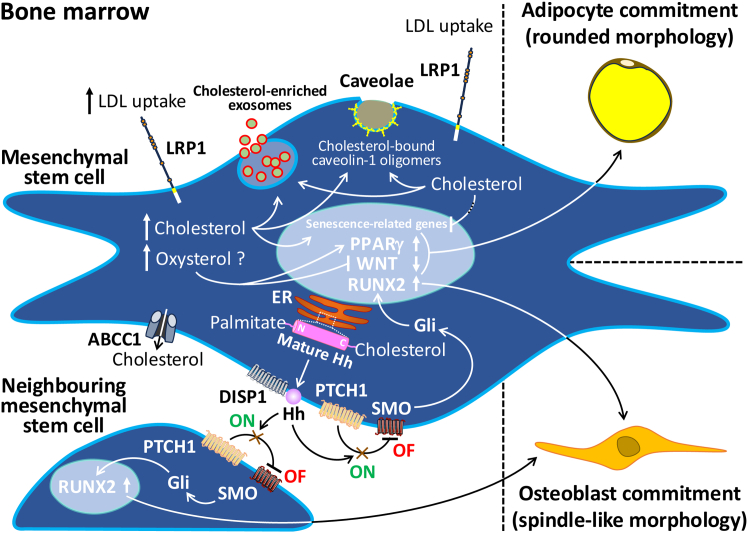
Simplified overview of the effects of cholesterol on a mesenchymal stem cell (MSC) after entering the bone marrow At high cholesterol uptake (LRP1 is the most representative of the LDL receptor family), some effects are related to adipogenic differentiation and senescence of MSCs. It is likely that the products of cholesterol oxidation are involved in adipocyte commitment via stimulation of PPARγ and repression of WNT signaling. With normal cholesterol uptake, some effects are related to homeostasis (caveolae) and rejuvenation (ROS/p53/p21^Cip1/Waf1^ signaling pathway), including those promoting autocrine and paracrine functions (exosomes) and osteoblastic differentiation of MSCs. As with the effect on senescence-related genes, the disruption of cholesterol-mediated benefits is expected to follow the increase in cytoplasmic cholesterol levels. Hedgehog formation with bound cholesterol and palmitic acid mediates a switch mechanism that allows activation of RUNX2 via SMO and Gli, leading to osteoblastic differentiation in the MSC (autocrine) or in neighboring MSCs (paracrine). Cholesterol can exit the cytoplasm through ABC transporters (ABCC1/ABC1). LRP1, LDL receptor-related protein-1; LDL, low-density lipoprotein; PPARγ, peroxisome proliferator-activated receptor γ; WNT, Wingless-related integration site; ROS, reactive oxygen species; RUNX2, runt-related transcription factor-2; ER, endoplasmic reticulum; Gli, glioma-associated oncogene; Hh, Hedgehog; DISP1, Dispatched-1 receptor; PTCH1, Patched-1 receptor; SMO, Smoothened receptor; ABC, adenosine triphosphate-binding cassette.

While excessive cholesterol intake and abnormally high blood cholesterol levels are often linked, other nutrients such as medium- and long-chain saturated fatty acids (palmitic acid, 16:0), may have an even greater effect on blood cholesterol levels,^57^ and, therefore, on the biology of BM-MSCs. In mouse models of high saturated fat diet-induced obesity, animals suffered from hypercholesterolemia, which was associated with increased cell death and cellular senescence of BM-MSCs compared with BM-MSCs from lean mice.^58^ Interestingly, cholesterol and palmitic acid have been documented as stable modifiers at the C- and N-terminal domains of protein ligands, including the transmembrane protein Dispatched-1, which is involved in the production and release of extracellular vesicles, of the Hedgehog signaling pathway in BM-MSCs.^59^ Cholesterol is added by an autoprocessing reaction, and palmitic acid is added by Hedgehog acetyltransferase. The proteins of the Hedgehog family are evolutionarily conserved morphogens with broad functional versatility, providing cells with positional information and fate guidance during early embryonic development and adult tissue regeneration.^60^^,^^61^ We reasoned that neighboring BM-MSCs that are not targeted by the endosomally controlled release of lipid-modified Hedgehog ligands (such as Sonic Hedgehog) in time may remain in an undifferentiated and quiescent adherent state (the Hedgehog pathway is “off”). However, when these ligands bind to the transmembrane cholesterol transporter Patched-1 on the surface of neighboring BM-MSCs that have been targeted, the seven-transmembrane transducer Smoothened is no longer inhibited, allowing osteoblastic differentiation to proceed via members of the cubitus interruptus-related Gli (glioma-associated oncogene) transcription factor family (the Hedgehog pathway is “on”). It is likely that non-classical Hedgehog signaling, through Gli-independent mechanisms, is responsible for the migration of newly formed osteoblasts or BM-MSCs in the process of becoming osteoblasts to the endosteal niche.^59^ The human Sonic Hedgehog has a uniquely high degree of specificity for cholesterol and palmitic acid but not for other fatty acids.^62^ The fact that both cholesterol and palmitic acid are exclusively involved in this sophisticated mechanism of regulation of the osteogenic differentiation of BM-MSCs suggests that both lipids are essential for the maintenance of bone and BM health (Figure 2). In model organisms, circulating triglyceride-rich lipoproteins (mainly derived from the gut) and extracellular vesicles have been shown to be able to transport Hedgehog containing covalently bound cholesterol and palmitic acid,^63^^,^^64^ opening up a wide world of possibilities, yet to be explored in mammals, to find lipid-activated Hedgehog anywhere to assist in emergency (acute) or restoration of morphogen signaling deficiencies (chronic) to maintain homeostasis or healing. It would be interesting to see whether the excess of cholesterol and palmitic acid in the BM, due to uncontrolled intake or metabolic dysfunction, might desensitize the Hedgehog pathway in BM-MSCs, thereby unbalancing their differentiation capacity and promoting the counteraction of adipogenic commitment. Another possibility is that, in the absence of binding by other fatty acids, they may interfere with the signaling pocket close to, or at least aligned on the same side of, the Hedgehog ligand for binding cholesterol and/or palmitic acid, thereby severely disrupting the Hedgehog pathway.^65^^,^^66^^,^^67^

To maintain a constant pool of BM-MSCs in the BM, it is plausible that prior to the above-mentioned steps of lipid-mediated differentiation of BM-MSCs into osteoblasts or adipocytes, BM-MSCs undergo asymmetric division to generate a stem cell capable of self-renewal and a daughter cell that acquires the commitment to differentiate, also to avoid the risk of long-lasting clonal expansion.^68^ In addition to cytoskeletal remodeling and membrane reorganization for migration plasticity,^69^ if the commitment of BM-MSCs is further indicative of osteoblast formation, it is also expected that their orientation and distribution from the central to the endosteal niche must be aligned and docked in an adherent state to the bone matrix. If the fate is to form BM adipocytes, the positioning of the progeny toward the BMAT, for which budding of daughter cells should occur, should result in the release of non-adherent, rounded, newly formed adipocytes or BM-MSCs in the process of becoming adipocytes. The correct control of this dual contrasting behavior of functional adherent spindle cells and non-adherent rounded cells derived from BM-MSCs may be of critical importance in the achievement of bone and BM homeostasis. This would be enriched by knowledge of whether the BM-MSC daughter cells undergo single-cell migration or collective-cell migration, which may depend on the structural and molecular composition of the surrounding extracellular matrix (such as gap size, stiffness, or orientation) in the BM niche during each of the differentiation processes,^69^ not excluding environmental signals (such as exogenous or endogenous lipids and their levels). In fact, recent advances point to lipid-mediated regulation of the Hedgehog pathway as a potential mechanism by which BM-MSCs not only dictate such a binary fate but also keep each differentiated cell type within the appropriate BM niche.^70^ This may be accomplished, at least in part, by the primary cilia of BM-MSCs, which could be retained in their progeny (osteoblasts and adipocytes), where the Hedgehog signaling pathway is found.^71^ In addition to human genetic disorders known as ciliopathies, in which the structure or protein composition of the cilium is defective,^72^^,^^73^^,^^74^ the lack of primary cilia in human BM-MSCs has been shown to be a barrier to their differentiation into osteoblasts or adipocytes.^75^^,^^76^ These unique and inconspicuous organelles, which include the microtubule-based axoneme, the ciliary membrane with its ciliary receptors, and the transition zone at the base of the cilium (which controls the localization of proteins to the ciliary membrane or the cytoplasm), are conserved in almost all modern eukaryotic cells. They play a key role in processing, mediating, and transducing mainly intercellular signals, whether they are chemical, physical, or biological.^74^^,^^77^^,^^78^ The cilium can house cholesterol, which is also the major component of the ciliary membrane, and fatty acids. Recent studies have underlined the significance of G protein-coupled receptor 120 (GPR120), also known as free fatty acid receptor 4 (FFAR4), in human BM-MSCs,^79^ which is located in the ciliary membrane near Hedgehog. Importantly, FFAR4 interacts with long-chain saturated fatty acids and long-chain unsaturated fatty acids, including ω-3 family fatty acids, which can induce adipogenic commitment in BM-MSCs after FFAR4 activation.^77^ Therefore, the convergence of target genes and the trafficking of specific proteins along the cilium as a result of the lipid-mediated activation of the Sonic Hedgehog and FFAR4 pathways may be translated into different properties and phenotypes of BM-MSCs, including those associated with cell morphology and modes of movement or attachment for proper orientation of polarity. Understanding the combined function of both signaling pathways may be invaluable to the biology of BM-MSCs in relation to cholesterol and fatty acid metabolism; indeed, it has been proposed that one pathway may be a fine-tuner of the other, probably due to allosteric effects between the lipids involved.^77^

In regenerative medicine, transplantation using BM-MCSs has some limitations in clinical applications, mostly due to poor survival, insufficient migration, and homing to the targeted tissue.^80^ The possible causes of this low efficacy in both allogeneic and autologous cell-based therapies are diverse and may be related to factors intrinsic or extrinsic to the receptor or donor. Among the intrinsic factors, age and sex are prominent, with clonogenic capacity and proliferation rate of BM-MSCs decreasing with age and in female donors in comparison to male donors.^81^ Among the extrinsic factors, the clonogenicity of BM-MSCs is suppressed in both male and female donors as the body mass index increases.^82^ Interestingly, the risk of MSC transplant failure has been shown to be significantly increased in receptor-obese patients with hypercholesterolemia, likely due to ROS-induced apoptosis mediated by the membrane ATP-binding cassette subfamily A member 1,^83^ establishing a potential role for adequate blood cholesterol levels and proper function of cholesterol efflux pathway-related proteins for the survival of circulating BM-MSCs prior to transplantation. This also suggests that cholesterol, either from exogenous or endogenous sources, may have different effects on the metabolism and behavior of BM-MSCs, depending on whether they are inside or outside the BM compartment. Note that BM-MSCs within the BM resting niche have a flat and presumably comfortable adherent morphology, whereas BM-MSCs that migrate from such an adhesive landscape and emerge from the BM have a rounded morphology, which is likely to be stressful as it is an unusual shape away from its natural environment. Therefore, it is reasonable to speculate that the differences in the geometry of BM-MSCs have an impact on their sensitivity in response to environmental cues.

## How fatty acids can affect MSCs in the BM

While it is clear that fatty acids, either from diet, liver, BM milieu and/or endogenous pathways, are essential for energy supply to BM-MSCs via the OXPHOS system, allowing versatile substrate utilization, their effects on the behavior and function of these cells in the BM are largely unknown. For example, it is uncertain whether, upon entering the BM via lipoprotein lipase,^19^ probably with the assistance of the syndecans^84^^,^^85^ (a small family of four transmembrane proteoglycans), circulating triglyceride-rich lipoproteins containing exogenous fatty acids activate and redirect resting BM-MSCs toward BM adipocytes, thereby initiating the lipid biogenesis program. Alternatively, they may be directly taken up and accumulated during the adipogenic differentiation of activated, detached BM-MSCs. This points to the need to investigate the signals that instruct BM-MSCs to leave their niche and expand BMAT in response to the increased fatty acid availability. A metabolic aspect recently highlighted was the potentially greater importance of triglycerides than that of cholesterol in fasting circulating lipoproteins for bone and BM health in apparently healthy adult men and women.^86^ At least in the normal range of the fasting state, triglycerides appeared to have a protective effect on bone turnover. Not without controversy due to conflicting data from other studies, this beneficial effect of fasting triglycerides on bone mass has also recently been reported in a postmenopausal cohort, using machine learning methods,^87^ and in a population-based study after re-analysis of data from the US National Health and Nutrition Examination Survey.^88^ It is important to note that the greatest (acute) influx of triglycerides under physiological conditions occurs in the fed state, when large amounts of chylomicrons with a fatty acid composition very similar to that of meals are transiently produced. However, the limits of blood triglyceride fluctuations during the day are not yet in place.^89^

A recent review has highlighted the association of plasma essential fatty acids (ω-6 and ω-3 families) with improved bone mineral density in women at risk of osteoporosis through a positive influence on osteoblastogenesis at the expense of osteoclastogenesis.^90^ However, after osteoblastogenic or adipogenic differentiation of healthy donor BM-MSCs in the presence of arachidonic acid (20:4ω-6), eicosapentaenoic acid (20:5ω-3), or docosahexaenoic acid (22:6ω-3), the ω-6 family fatty acid was pro-adipogenic, whereas the ω-3 family fatty acids were pro-osteoblastogenic,^91^ consistent with compelling evidence from preclinical and epidemiological studies of the beneficial effects of ω-3 polyunsaturated fatty acids on bone health, including in aging.^92^ Contrasting effects of ω-6 and ω-3 fatty acids have been demonstrated in a mouse model of obesity induced by an oil rich in linoleic acid (18:2ω-6) supplemented with eicosapentaenoic and docosahexaenoic acids.^93^ Fatty acids from the ω-3 family counteracted the negative effects of linoleic acid-induced changes in BM-MSCs, promoting osteoblastogenic commitment and reducing senescent markers. At the mechanistic level, linoleic acid has been shown to suppress osteoblastogenesis *in vitro* by attenuating the expression of runt-related transcription factor 2 and osterix in murine BM-MSCs.^94^ In addition, an outstanding study has described the interplay of long-chain ω-3 fatty acids surrounding BM-MSCs and the biophysical adaptation of the cytoplasmic membrane of BM-MSCs in the process of BM-MSC differentiation into osteoblasts or adipocytes.^95^ Supplementation of undifferentiated human BM-MSCs with docosahexaenoic acid resulted in an increase in this fatty acid and eicosapentaenoic acid, instead of ω-6 fatty acids, in the specific lipid class of phosphatidylethanolamine plasmalogen, similar to the lipid distribution after osteoblast differentiation. In combination with the promotion of osteoblastic fate through the serine/threonine kinase Akt (also known as protein kinase B) signaling, these lipid-cytoplasmic membrane changes were potentially associated with the phenotypic plasticity of BM-MSCs. This may be related to the cytoskeletal remodeling and membrane reorganization required for the differentiation and mobilization of BM-MSCs from their quiescent niche.

The basis for the possible *in vivo* differences in the type of non-essential fatty acids on the BM-MSCs has already been established by lipidomics of BM aspirates from different vertebral localizations in young volunteers.^96^ With hardly any differences between the various vertebrae, the most common fatty acids were monounsaturated fatty acids (mainly oleic acid, 18:1ω-9), followed by saturated fatty acids (mainly palmitic acid). Palmitic acid is the most abundant saturated fatty acid, while oleic acid is the most abundant monounsaturated fatty acid in the body and in the diet, including in other orders of nature. Previous indirect *in vitro* studies have shown that stearic acid (18:0) and palmitic acid, probably through a feedback inhibitory mechanism on fatty acid synthase, but not excluding others,^97^^,^^98^ reduce proliferation and function of and increase apoptosis in osteoblasts cultured from the spongy BM of young volunteers.^99^ Palmitic acid also promoted BM adipocytes by upregulating the adipogenic transcription factors PPARγ and CCAAT enhancer-binding protein α and downregulating the osteogenic transcription factors runt-related transcription factor 2 and distal-less homeobox 5 in BM-MSCs from patients with osteonecrosis of the femoral head.^100^ In contrast, oleic acid not only did not show these effects in iliac crest BM-MSCs from osteonecrosis patients or healthy donors or in osteoblasts derived from these cells but also ameliorated the adverse effects of palmitic acid-induced cell death.^101^^,^^102^ Overexpression in BM-MSCs *in vitro* and *in vivo* of the endoplasmic reticulum membrane-bound protein stearoyl-CoA desaturase 1, which catalyzes the conversion of palmitic acid and stearic acid to palmitoleic acid (16:1ω-7) and oleic acid, respectively, has recently been shown to have similar effects.^103^ It is tempting to speculate that palmitic acid and oleic acid are part of a metabolic rheostat that controls the commitment of BM-MSCs to differentiate into adipocytes or osteoblasts; if the BMAT needs to expand, then palmitic acid may work to promote BM adipogenesis, and if the bone needs to increase the osteoblast/osteoclast balance, then oleic acid may help to promote osteoblastogenesis. In support of this, BM-MSCs from the femur of non-osteoporotic and osteoporotic individuals accumulate oleic acid and palmitic acid, respectively, in the plasma membrane as they differentiate into osteoblasts,^104^ suggesting that priming BM-MSCs with oleic acid, but not palmitic acid, enhances their ability to maintain bone homeostasis. Preconditioning of BM-MSCs by chemical or physical methods is currently a promising strategy to overcome survival, migration, and homing limitations in clinical applications.^105^^,^^106^^,^^107^ A representative recent example is the *ex vivo* pre-treatment of BM-MSCs with valproic acid (2-propylpentanic acid) for *in vivo* remyelination of the corpus callosum in a mouse model of cuprizone-induced demyelination.^108^ Although it is too early to extrapolate these results to potential effects of palmitic acid- or oleic acid-rich diets on the biology of human BM-MSCs, there is increasing evidence to suggest this. In animal studies, obesity induced by a high-saturated fat diet reduced the stemness of BM-MSCs and made BM-MSCs prone to differentiate into adipocytes, leading to the expansion of BMAT and a decrease in bone mass^109^—effects of BM-MSC aging that may be mediated by histone lysine demethylase 4B,^110^ suggesting epigenetic mechanisms. The most recent approximation to the *in vivo* effect of dietary oleic acid on human BM-MSCs and/or bone-forming cells and/or bone-resorbing cells is an adult cross-sectional study showing a positive association between bone health and daily consumption of approximately 20 g of monounsaturated fatty acids (mainly oleic acid).^111^ Less implicit studies based on the consumption of the Mediterranean diet, whose main components include olive oil (the most important natural dietary source of oleic acid) and some foods rich in ω-3 fatty acids, have also shown that adherence to this diet reduces fracture risk in various populations worldwide.^112^^,^^113^ Therefore, it can be inferred that there is no better way to precondition BM-MSCs *in vivo* in a natural way to give them the programming capacity than by consuming a balanced diet that is rich in oleic acid and moderate in ω-3 fatty acids. Because of its potential role in BM-MSCs and its many other health benefits,^114^^,^^115^^,^^116^^,^^117^^,^^118^ olive oil and sources of ω-3 fatty acids can be dietary choices to effectively manage BM-MSCs, protect the BM, and promote human wellbeing.

Another novel aspect recently discovered is the link between energy metabolism and the adipogenic commitment of human BM-MSCs, based on a mutually exclusive competition of the OXPHOS system and the glycolytic pathway. When the OXPHOS system is activated while the glycolytic pathway is inhibited *in vitro*, BM-MSCs are induced to differentiate into adipocytes,^119^ suggesting that fatty acids, as natural inducers of the OXPHOS system,^120^ may act as part of the signaling network in the BM to activate BM-MSCs and initiate and feedback the expansion of BMAT. This dissociated metabolic reprogramming of the mitochondrial energetics has also been described to be cholesterol dependent in human stem-like glioma spheres, shifting to activation of the OXPHOS system for resistance to ferroptosis,^121^ and in several human cancer cells, shifting to activation of aerobic glycolysis followed by lactic acid fermentation (Warburg effect) for rapid cell proliferation.^122^ The mechanistic effect of endogenous or exogenous cholesterol, alone or in combination with fatty acids, on the behavior and fate of BM-MSCs in terms of their bioenergetics, which could also clearly compromise their therapeutic utility, is a topic for future research.

While intracellular lipid levels can be increased endogenously via *de novo* lipogenesis, the class B scavenger receptor CD36/fatty acid translocase has been shown to play an important role in the internalization of exogenous fatty acids and lipoproteins, with implications for the adipocytic commitment of human BM-MSCs.^123^ Mechanistically, CD36 promotes the uptake of fatty acids, enabling carnitine palmitoyl-transferase 1A (CPT1A) to transport fatty acyl chains from the cytosol into the mitochondria. Excessive uptake of fatty acid and accelerated intracellular transport to the mitochondria have recently been shown to cause changes in the cell cycle (decreased proliferation and increased apoptosis) and differentiation (decreased multipotency) in BM-MSCs from patients with myelodysplastic syndrome.^124^ Interestingly, reduced intracellular transport of fatty acids to the mitochondria due to a reduction in CPT1A also causes BM-MSCs from healthy donors to enter in senescence, as in aging, with a slowing of their proliferation rate and a block in their ability to differentiate; when normal CPT1A levels are restored, CPT1A stabilizes ROS-scavenging proteins (superoxide dismutase 2), facilitates the distribution and fate of BM-MSCs toward osteoblast formation.^125^ Therefore, consistent with its role in fatty acid processing in mitochondrial redox hubs, fine-tuning of CPT1A may have a critical direct effect on preventing ROS overproduction and maintaining mitochondrial homeostasis in BM-MSCs. This underlines the critical role of the tight control of exogenous fatty acid supply and its coupling with endogenous fatty acid utilization in the metabolic and functional activities of BM-MSCs and suggests that CPT1A may be a relevant marker to establish the health and therapeutic utility of these cells.

At a different level of regulation, the symmetric division of BM-MSCs enables emergency responses to tissue damage from the BM. In the symmetric division for mobilization, which can function as symmetric self-renewal to maintain the niche, the mode of division implies a progeny cell that is genetically and phenotypically identical to the original stem cell.^68^ Theoretically, this progeny is not primed to immediately adopt a fate. Clonal BM-MSCs are then rapidly mobilized into the bloodstream to homing in on sites of injury, where they create the regenerative conditions for wound healing.^9^^,^^126^ Despite the prediction of endogenous or exogenous transient factors that act in an injury-inducible manner to mobilize BM-MSCs into the circulation, this issue is barely understood. Recent studies have highlighted the role of endocannabinoids derived from the endogenous metabolism of dietary saturated, monounsaturated, and polyunsaturated fatty acids, such as 2-arachidonoylglycerol (2-AG), *N*-docosahexaenoylethanolamine (DHEA), and *N*-palmitoylethanolamide (PEA), as putative key lipid factors in inducing BM-MSCs to leave the BM via activation of cannabinoid receptors (most likely cannabinoid receptor 2 [CB_2_R]).^127^^,^^128^^,^^129^ Endocannabinoids have been implicated in the modulation of many physiological processes, including adipogenesis (as Smoothened inhibitors),^63^ and pathophysiological conditions, including inflammation.^130^ The endocannabinoid system comprises the endocannabinoids themselves and cannabinoid receptors and proteins involved in the biosynthesis, transport, and resolution of cannabinoid activity, such as the enzymes fatty acid amide hydrolase coupled to monoacylglycerol lipase and *N*-acylethanolamine-hydrolyzing acid amidase or α/β-hydrolase domains 6 and 12.^131^^,^^132^ Endocannabinoids can be generated not only in the brain and peripheral tissues but also in the BM. Under stress, human BM-MSCs can release endocannabinoids to increase the BM endocannabinoid pool, which may act as a paracrine mechanism to mobilize HSCs and other hematopoietic cells with high levels of CB_1_R and CB_2_R expression.^133^ In animal models of injury, β3 adrenergic agonists have recently been shown to increase local BM levels of endocannabinoids and their congeners through a mechanism of fatty acid amide hydrolase inhibition, which promotes the mobilization of BM-MSCs.^134^ The high-mobility group protein B1 and the neuropeptide substance P are known to induce BM-MSCs to migrate out of the BM,^135^^,^^136^ and both have recently been shown to be regulated by endocannabinoids.^137^^,^^138^ Therefore, endocannabinoids can be placed at the head of a family of fatty acid-dependent lipid metabolites that are pam mobilizers of BM cells. Whether the production and release of endocannabinoids by BM-MSCs are part of an autocrine regulatory loop that helps to balance their traffic from the BM to meet internal or external demands is a question that needs to be investigated. Further investigation into the interaction of endocannabinoids with other factors involved in the mobility of BM-MSCs is also needed. It will also be interesting to study whether endocannabinoids participate in osteoblastic or adipogenic differentiation by interfering with Smoothened in the Sonic Hedgehog pathway of BM-MSCs in the BM or extramedullary sites.

**Figure 3:**
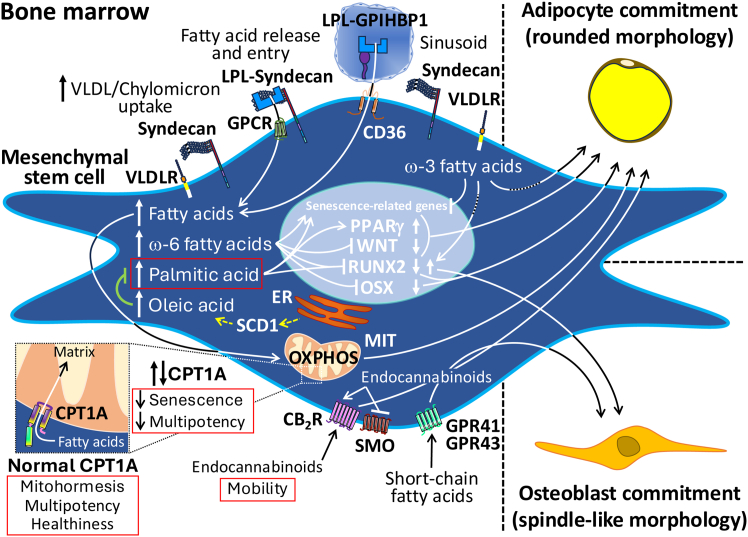
Simplified overview of the effects of fatty acids on a mesenchymal stem cell (MSC) after they enter the bone marrow Fatty acids can be internalized as triglyceride-rich lipoproteins (VLDL and chylomicrons) by receptors (VLDLR) and heparan sulfate proteoglycans (syndecans), and as free fatty acids from the extracellular to the intracellular milieu (GPCRs and CD36) after the action of LPL (dimers) bound to syndecans on the MSC surface and to GPIHBP1 on the capillary endothelium. At high fatty acid uptake, some effects of ω-6 fatty acids (even at normal uptake) are related to the adipogenic differentiation (by stimulation of WNT and repression of RUNX2 and OSX signaling) and senescence of MSCs. At normal fatty acid uptake, some effects of ω-3 fatty acids are related to rejuvenation and osteoblastic differentiation (PKB signaling pathway), but also to the adipogenic differentiation (FFAR4 signaling pathway) of MSCs. Some effects of palmitic acid (at normal or high uptake) are related to the adipogenic differentiation (through stimulation of PPARγ and C/EBPα and repression of RUNX2 and DLX5 signaling). Some effects of oleic acid (at normal or high uptake) are related to the opposite and preventive effects of palmitic acid, including palmitic acid-induced MSC death; these are mimicked in MSCs overexpressing SCD1, which catalyzes the formation of oleic acid. Increased fatty acid uptake also drives the MSC mitochondrial OXPHOS machinery, which induces adipocyte commitment. Uncoordinated entry of fatty acids into the mitochondrial matrix due to increased or decreased activity of CPT1A induces senescence and inhibits multipotency in MSCs. However, normal levels (activity) of CPT1A allow the acquisition of MSC mitohormesis, multipotency, and health. Endocannabinoids in the cytoplasm from MSC fatty acid metabolism induce adipocyte commitment (by stimulating CB_2_R and inhibiting SMO). Some effects of endocannabinoids from surrounding cells in the BM milieu are related to the stimulation of CB_2_R, which induces the mobility of the targeted MSC. Some effects of microbiota-producing SCFAs are related to the osteogenic differentiation (through stimulation of GPR41 and GPR43 as well as GPR41- and GPR43-independent pathways). For clarity, C/EBPα, DLX5, PKB, and FFAR4, among others, are not shown. VLDL, very-low density lipoprotein; VLDLR, VLDL receptor; GPCRs, G-protein coupled receptors; CD36, class B scavenger receptor cluster of differentiation 36 (CD36)/fatty acid translocase; LPL, lipoprotein lipase; GPIHBP1, glycosylphosphatidylinositol-anchored high-density lipoprotein-binding protein 1; RUNX2, runt-related transcription factor-2; OSX, osterix; PKB, protein kinase B; FFAR4, free fatty acid receptor 4; PPARγ, peroxisome proliferator-activated receptor γ; C/EBPα, CCAAT enhancer-binding protein α; DLX5, distal-less homeobox 5; SCD1, stearoyl-CoA desaturase-1; OXPHOS, oxidative phosphorylation; MIT, mitochondrion; CPT1A, carnitine palmitoyl-transferase 1 A; CB_2_R, cannabinoid receptor 2; SMO, Smoothened receptor; SCFAs, short-chain fatty acids; GPR41, G protein-coupled receptor 41; GPR43, G protein-coupled receptor 43.

It is worth noting that the relationship between gut microbiota and bone metabolism is being more widely understood.^139^^,^^140^^,^^141^ The gut microbiota is a complex, mutualistic, symbiotic ecosystem comprising a vast array of microorganisms. It is shaped by a combination of deep ecological and evolutionary origins in relation to the host and serves to adapt to and benefit from environmental influences resulting from food consumption. Short-chain (saturated) fatty acids (SCFAs), consisting mainly of acetic (2:0), propionic (3:0) and butyric (4:0) acids, are some of the most important gut microbial products resulting from the anaerobic fermentation of dietary fiber-rich, indigestible carbohydrates.^142^ Virtually all (>90%) of the SCFAs are produced by the microbiota in the gut, where acetic acid, propionic acid, and butyric acid are found in a 3:1:1 ratio, while a small proportion comes from the diet or is formed during the metabolism of proteins.^143^ SCFAs can enter the host (acetic acid is the most abundant SCFA in plasma) and reach the complex metabolic pathways that regulate the osteoblastogenesis-osteoclastogenesis axis by improving bone mineralization, which may have clinical implications in the prevention and treatment of osteoporosis.^144^ In addition, previous studies have shown that BM-MSCs lose their stemness in a germ-free mouse model and that their ability to proliferate and differentiate is normalized after their recolonization in the gut microbiota,^145^ suggesting that the gut microbiota, in tandem with diet, may be important in regulating the biology of BM-MSCs. In support of this idea, more recent studies have demonstrated that GPR 41 and 43 for SCFAs promote trabecular bone mass by promoting osteoblastogenesis in the bones of wild-type mice fed a chow diet.^146^ However, when the animals were fed on a diet containing 10% inulin, which markedly increased the production of microbial SCFAs (particularly acetic acid) in the portal circulation, bone mass further increased in GPR41/43-deficient mice, while BM adiposity was blunted, with no differences between GPR41/43-deficient mice and wild-type littermates. This suggests that SCFAs have little, if any, adipogenic activity on BM-MSCs and that BM-MSCs can be induced to differentiate into osteoblasts by SCFAs through GPR41- and GPR43-dependent and -independent mechanisms (Figure 3). As an example of mutualism, the transplantation of (healthy) human umbilical cord-MSCs for therapeutic benefit in a mouse model of systemic lupus erythematosus could increase the abundance of butyric acid-producing microbiota (*Lactobacillus johnsonii* and *Romboutsia ilealis*) in the gut, which attenuated disease progression through a mechanism of butyric acid interaction linked to the aryl hydrocarbon receptor transcription factor.^147^ In line with this, defective BM-MSCs in patients with systemic lupus erythematosus can be therapeutically modulated by aryl hydrocarbon receptor antagonists.^148^ Abnormal aryl hydrocarbon receptor activation was shown to suppress proliferation and osteoblast maturation via inhibition of β-catenin in BM-MSCs from a mouse model of collagen-induced arthritis.^149^ This suggests the potential role of the aryl hydrocarbon receptor pathway, perhaps at the intersection of other lipid-induced pathways,^150^ as a target for SCFAs in improving the health status of BM-MSCs in terms of stemness and multipotency.

## Conclusions and future perspectives

The importance of the lipids, particularly cholesterol and fatty acids, in the biology of BM-MSCs has rarely been overemphasized. Even less work has been done to distinguish between the endogenous and exogenous origins of these lipids. However, every time we eat fats, the lipids from these fats are absorbed, and their levels rise in the blood, reaching every part of the body. The type and the amount of lipids during these metabolic fluctuations may influence both circulating BM-MSCs (on their way to regenerate injured tissue) and static BM-MSCs (resident in their BM niche and engaged in their mission to maintain the healthy balance of BM cells). This includes systemic lipid metabolism and specific endogenous lipid metabolism in the BM, collectively having long-term regulatory effects on BM-MSCs.

From the point of view of a healthy diet to protect the BM from physiological or pathophysiological damage, it can also be concluded that there must be a fine control of cholesterol and fatty acids in the BM, as they are able to regulate different, even opposing effects on the biology of BM-MSCs, depending on the mechanisms in which they are involved (Table 1). The hypermorphic nature of the master *PPARγ* gene, with the bone and BMAT in the middle, is a striking example of this kind of extremely subtle control.^151^ Interestingly, BMAT can compartmentalize and process these lipids in a spatially and temporally regulated manner, probably as a function of stochastic events and biological noise in the BM microenvironment, among other factors.

**Table 1:** Effects and mechanistic insights into fatty acids and cholesterol on the function and behavior of bone marrow mesenchymal stem cells

	Description	Reference
**Function**		

Maintain descendant cells (adipocytes and osteoblasts) in their niche	by exogenous/endogenous cholesterol and palmitic acid (SFAs); mechanisms involving the Hedgehog signaling pathway; in normal/healthy conditions	Ma et al.^70^ Petsouki et al.^71^
Decrease regenerative properties	by exogenous/endogenous cholesterol; mechanisms involving the ROS/membrane ABCA1 signaling pathway; in obesity accompanied by hypercholesterolemia	Kim et al.^83^
Increase regenerative properties	by endocannabinoids from exogenous SFAs, MUFAs, and PUFAs; mechanisms involving the CB2R, FAAH, HMGB1, and neuropeptide substance P signaling pathways; in normal/healthy conditions	Girousse et al.^127^ Casati et al.^128^ Lopez-Gonzalez et al.^129^ Xie et al.^135^ Tao et al.^136^ Abdallah et al.^137^ Greco et al.^138^
Mobilize hematopoietic stem cells (HSCs)	by endocannabinoids from exogenous SFAs, MUFAs, and PUFAs; mechanisms involving the CB1R and CB2R signaling pathways; in normal/healthy conditions	Köse et al.^133^

**Behavior**		

Differentiate into adipocytes	by exogenous cholesterol; mechanisms involving the PPARγ and WNT signaling pathways; in both normal/healthy conditions and in steroid-induced osteonecrosis	Rendina-Ruedy et al.^33^ Wang et al.^41^ Wu et al.^46^
	by exogenous long-chain ω-3 fatty acids (PUFAs); mechanisms involving the GPR120/FFAR4 signaling pathway; in normal/healthy conditions	Hilgendorf et al.^77^ Wang et al.^79^
	by exogenous long-chain ω-6 fatty acids (PUFAs); mechanisms involving the RUNX2/osterix signaling pathway; in normal/healthy conditions, at risk of osteoporosis and obesity	Das^90^ Casado-Diaz et al.^91^ Benova et al.^93^ Nesbeth et al.^94^
	by exogenous/endogenous palmitic acid**;** mechanisms involving the PPARγ/C/EBPβ and RUNX2/DLX5 signaling pathways; in normal/healthy conditions and osteonecrosis	Elbaz et al.^99^ Gillet et al.^100^
	by exogenous SFAs; mechanisms involving the class B scavenger receptor CD36/CPT1A signaling/metabolic pathway; in overfeeding conditions	Dawodu et al.^123^
	by endocannabinoids from exogenous SFAs, MUFAs, and PUFAs; mechanisms involving the SMO signaling pathway; in normal/healthy conditions	Prince et al.^63^
Delay their aging	by exogenous cholesterol; mechanisms involving the ROS/p53/p21^Cip1/Waf1^ signaling pathway and autophagy processes; in senescent conditions	Zhang et al.^56^
Accelerate their aging	by endogenous cholesterol; mechanisms involving the metabolism of SFAs; in overfeeding conditions	Sagar et al.^58^
Differentiate into osteoblasts	by exogenous/endogenous cholesterol; mechanisms involving the Hedgehog signaling pathway; in normal/healthy conditions	Wang et al.^59^
	by exogenous/endogenous palmitic acid; mechanisms involving the Hedgehog signaling pathway; in normal/healthy conditions	Wang et al.^59^
	by exogenous long-chain ω-3 fatty acids; mechanisms involving changes in membrane phosphatidylethanolamine plasmalogen through the serine/threonine kinase Akt (PKB) signaling pathway; in normal/healthy conditions, obesity, and aging	Das^90^ Casado-Diaz et al.^91^ Ali et al.^92^ Benova et al.^93^ Levental et al.^95^
	by exogenous/endogenous oleic acid (MUFA); mechanisms involving the counteraction of palmitic acid-derived effects on the PPARγ/C/EBPα/and RUNX2/DLX5 signaling pathways; in normal/healthy conditions and osteonecrosis	Gillet et al.^101^ Fillmore et al.^102^ Fang et al.^111^
	by endogenous short-chain SFAs; mechanisms involving the GPR41 and GPR43 signaling pathways; in normal/healthy conditions	Behler-Janbeck et al.^146^
Decrease their stemness	by exogenous SFAs; mechanisms involving the histone KDM4B; in overfeeding conditions	Tencerova et al.^109^ Deng et al.^110^
Decrease their proliferation and multipotency and increase their apoptosis	by exogenous SFAs; mechanisms involving the CD36/CPT1A signaling/metabolic pathway; in myelodysplastic syndrome accompanied by overfeeding	Yin et al.^124^
Increase their stemness	by endogenous short-chain SFAs; mechanisms involving the GPR41 and GPR43 signaling pathways; in normal/healthy conditions	Xiao et al.^145^

SFAs, saturated fatty acids; MUFAs, monounsaturated fatty acids; PUFAs, polyunsaturated fatty acids; ROS, reactive oxygen species; ABCA1, ATP-binding cassette subfamily A member 1; CB2R, cannabinoid receptor 2; FAAH, fatty acid amide hydrolase; HMGB1, high-mobility group protein B1; CB1R, cannabinoid receptor 1; PPARγ*,* peroxisome proliferator-activated receptor *γ*; WNT, Wingless-related integration site; GPR120, G protein-coupled receptor 120; FFAR4, free fatty acid receptor 4; RUNX2, runt-related transcription factor 2; C/EBPα, CCAAT enhancer-binding protein α; DLX5, distal-less homeobox 5; CD36, cluster of differentiation 36; CPT1A, carnitine palmitoyl-transferase 1A; SMO, Smoothened receptor; PKB, protein kinase B; KDM4B, lysine demethylase 4B.

Therefore, further research into how exogenous and endogenous lipids function in the intracellular and intercellular communications, particularly within the BM, may help elucidate the general principles for the effects of dietary lipids (which profoundly affect whole-body lipid metabolism) on the canonical characteristics of BM-MSCs. This integrative research holds promise for the development of metabolic strategies for the treatment of BM-related diseases or tissue regeneration.

## Acknowledgments

This work was supported by grant PID2022-139124NB-I00 funded by MICIU/10.13039/501100011033AEI/10.13039/501100011033 and by “ERDF A way of making Europe”. B.G.-G. is the recipient of grant PREP2022-000411 funded by MICIU/10.13039/501100011033AEI/10.13039/501100011033 and “ESF Investing in your future.” The funders had no role in study design, data collection, data analysis and interpretation, or drafting of the manuscript.

## Author contributions

Conceptualization, B.G.-G., F.J.G.M., and S.M.J.-C.; literature collection, B.G.-G., M.P., M.F., C.A., R.A., F.J.G.M., and S.M.J.-C.; investigation, B.G.-G., F.J.G.M., and S.M.J.-C.; writing – original draft, F.J.G.M.; writing – review & editing, B.G.-G., M.P., M.F., C.A., R.A., F.J.G.M., and S.M.J.-C.; graphical presentation, F.J.G.M.; supervision, F.J.G.M. and S.M.J.-C.; project administration, S.M.J.-C. funding acquisition, F.J.G.M. and S.M.J.-C.

## Declaration of interests

There are no competing interests to declare.
